# Host–virus interactions during infection with a wild-type ILTV strain or a glycoprotein G deletion mutant ILTV vaccine strain in an *ex vivo* system

**DOI:** 10.1128/spectrum.01183-24

**Published:** 2025-01-13

**Authors:** Gayathri Gopakumar, Mauricio J.C. Coppo, Andrés Diaz-Méndez, Carol A. Hartley, Joanne M. Devlin

**Affiliations:** 1 Asia-Pacific Centre for Animal Health, Melbourne Veterinary School, Faculty of Science, The University of Melbourne, Victoria, Australia; 2 Escuela de Medicina Veterinaria, Universidad Andrés Bello, Concepción, Biobío, Chile; Oklahoma State University College of Veterinary Medicine, Stillwater, Oklahoma, USA

**Keywords:** immune-evasion, host–virus interaction, immune response, interferon, gene enrichment, avian alphaherpesvirus, transcriptome, RNA-seq, *ex vivo*, tracheal organ culture (TOCs), explant, infectious laryngotracheitis virus (ILTV)

## Abstract

Previous studies have demonstrated the safety and efficacy of a live-attenuated glycoprotein G (gG) deletion mutant vaccine strain of ILTV (∆gG-ILTV). In the current study, transcriptional profiles of chicken tracheal organ cultures (TOCs), 24 h post inoculation with ∆gG-ILTV or the gG-expressing parent wild-type strain, CSW-1 ILTV were explored and compared with the mock-infected TOCs using RNA-seq analysis. Transcriptomes of the vaccine and wild-type ILTV were also compared with each other. Although no viral genes (except for gG) were differentially regulated between the two ILTV-infected TOCs, pair-wise comparison of the transcriptomes of the ∆gG-ILTV or the CSW-1 ILTV-infected TOCs (each compared with mock-infected TOCs) identified the similarities and differences in host gene transcription between them. Several immune checkpoint inhibitors with likely roles in ILTV-mediated immune augmentation, and gene ontologies indicating cytokine response, and cytokine signaling were upregulated in both TOCs. Additionally, several other biological processes, molecular functions, and cellular components were enriched uniquely in the ∆gG-ILTV-infected TOCs, including those that indicated modifications to tracheal extracellular matrix (ECM) structural components, which may have a role in immune modulation *in vivo*. This study has revealed that the modifications of transcription of host genes during the early stages of ILTV infection are not limited to changes in cytokine or chemokine gene transcription, but several other immune-related genes and ECM components. Moreover, their differential regulation in the *ex vivo* system appears to be influenced by gG expression, potentially affecting the outcome of ILTV infection *in vivo*.

**IMPORTANCE:**

Infectious laryngotracheitis virus (ILTV) remains a serious threat to poultry industries worldwide, causing significant economic losses. The glycoprotein G (gG) of ILTV is a virulence factor and a chemokine-binding protein with immunoregulatory functions. The influence of gG on the transcription of select host chemokine and cytokine genes has been demonstrated previously. This study extends our understanding of the early and localized host–ILTV interactions using genome-wide transcriptome analysis of ILTV-infected chicken tracheal organ cultures, and the role of gG during the process. Differential regulation of genes encoding immune checkpoint inhibitors observed in this study may have a role in ILTV-induced inhibition of type I interferon response, or negative regulation of T cell responses, bringing clarity to these ILTV immune-evasion mechanisms. Furthermore, differential regulation of genes encoding certain structural components and receptors with roles in cell migration, in the absence of gG, is consistent with the immunomodulatory role of ILTV gG.

## INTRODUCTION

*Iltovirus gallidalpha 1*, commonly known as infectious laryngotracheitis virus (ILTV), is the etiological agent of infectious laryngotracheitis (ILT), a contagious respiratory disease of poultry (1–3). The virus replicates in the tracheal and conjunctival epithelial cells during lytic infection (4) and establishes lifelong latency in trigeminal ganglia and tracheal tissues (4–6). Reactivation of latent virus during periods of stress, or horizontal transmission of the virus from infected to naïve or non-vaccinated flocks through aerosols and expectorants, results in ILT outbreaks (7). Outbreaks resulting from infection with viral strains that emerged from natural recombination between live-attenuated vaccines have also been reported (8). The disease is distributed worldwide, particularly in regions where there is intensive poultry production, causing morbidity, mortality, and decreased egg production, resulting in significant economic losses (9).

Understanding host–pathogen interaction is vital to decipher the molecular basis of infectious diseases such as ILT and for the development of effective vaccines and antivirals (10). With this goal in mind, several infection model systems that enabled the study of ILTV–host interactions have been explored in recent years (11–18). Each of these systems has its own advantages and disadvantages (19). While *in vivo* systems are considered the most reliable or relevant systems, inter-animal variations and influence of environmental factors are the major drawbacks. Although these limitations are overcome by *in vitro* infection models such as primary and continuous cell culture systems, they are devoid of three-dimensional architecture, maintained by cell-to-cell and cell-to-extracellular matrix (ECM) component interactions (19). Modifications to ECM composition and structure during pathogen infection have a profound impact on the outcome of infection (20), and as such, organ cultures or *ex vivo* systems make excellent alternatives for the study of host–pathogen interactions.

Reddy et al. found that the replication patterns of ILTV in tracheal organ cultures (TOCs) and conjunctival organ cultures were comparable to those observed *in vivo,* confirming the ability of these mucosal explants to mimic host physiological conditions (21) and hence their suitability for host–ILTV interaction studies. Using glycoprotein G (gG)-deficient (∆gG-ILTV) and glycoprotein-G-expressing strains of ILTV in different *in vitro*, *ex vivo,* and *in vivo* infection models, Coppo et al. demonstrated the influence of gG in the transcription of select cytokine and chemokine genes, underscoring the impact of this glycoprotein in inflammation, virulence, and balance of immune responses (17) during different stages of host response to ILTV. Earlier studies suggested that lack of viral chemokine-binding protein (vCKBP) gG during infection with ∆gG-ILTV appeared to modulate host immune response from a nonprotective humoral response toward a protective cell-mediated immune response (22–26), proposing the suitability of ∆gG-ILTV as a vaccine candidate for the control of ILT (27–29). Over the years, several studies have been conducted to demonstrate the protective efficacy and safety of this vaccine strain (30–34).

The aim of the current study was to expand our understanding of the localized response to ILTV infection in the trachea and decipher the role of gG during this process. Transcriptome-level differences between infection with the ∆gG-ILTV and the parent wild-type CSW-1 ILTV in TOCs were investigated and compared using RNA-seq analysis. The establishment of infection and ILTV replication in TOCs were also evaluated to assess the progress of infection over time.

## RESULTS

### Evidence of ILTV replication and localization in TOCs

**FIG 1:**
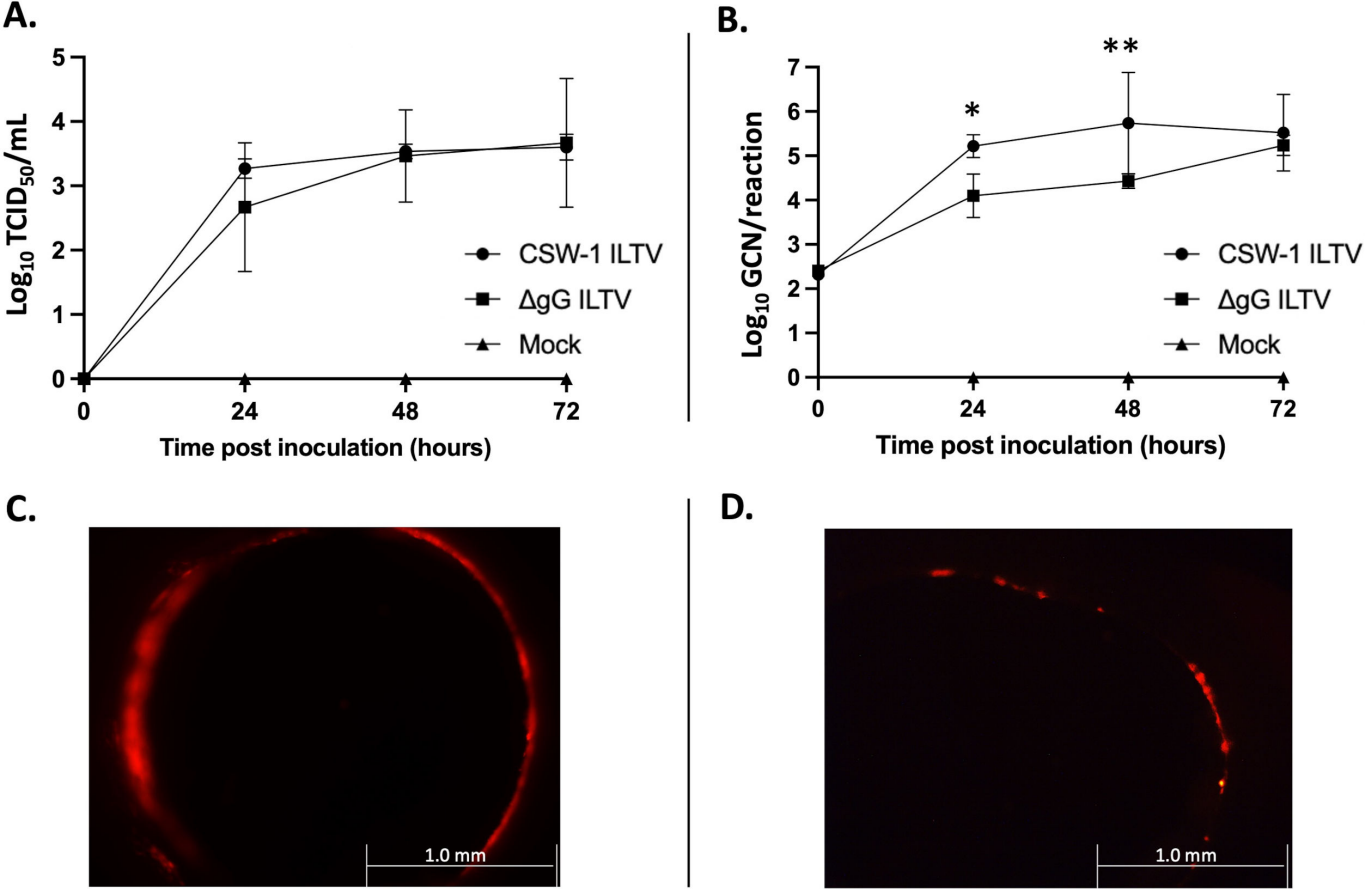
Establishment of ILTV infection and replication in TOCs. (**A**) TCID_50_ assay and (**B**) ILTV UL15 qPCR assay of supernatants collected from three replicates each of the mock-infected, CSW-1 ILTV-infected or ∆gG-ILTV-infected TOCs. The log_10_ mean titers and log_10_ mean GCNs are plotted in the graphs (A) and (B), respectively. Log_10_ TCID_50_/mL values and log_10_ GCN/reaction values of 0 were adjusted to 1 to allow graphing of log_10_ values. Error bars indicate standard deviation. Significant differences (*P*_adj_ < 0.05) in GCNs are shown with asterisks (*P_adj_ value = 0.0021 and **P_adj_ value = 0.0004). Fluorescent micrograph of tracheal rings captured at (**C**) 24 hpi and (**D**) 72 hpi with m-cherry ILTV (m-cherry fluorescent protein-tagged CSW-1 strain of ILTV), showing localization of m-cherry fluorescence. Scale bars in (**C**) and (**D**) indicate 1.0 mm.

Supernatant samples subjected to tissue culture infective dose (TCID_50_) assay and ILTV UL15 qPCR assay indicated active replication of ILTV in both the CSW-1 ILTV and ∆gG-ILTV-infected TOCs, but not in the mock-infected TOCs (Fig. 1A and B). Viral titers and ILTV genome copy numbers (GCNs) for both strains increased from 0 to 72 h post infection (hpi). TOCs infected with m-cherry ILTV (a different laboratory CSW-1 strain of ILTV that encodes m-cherry fluorescent protein) showed localization of red fluorescence in the tracheal epithelial cell lining from 24 hpi onward that diminished toward the end of the study period (72 hpi) (Fig. 1C and D). Light microscopy of TOCs revealed ciliary movement in the tracheal epithelial cells of the ILTV-infected (CSW-1 ILTV or ∆gG-ILTV) TOCs that was comparable to that of the mock-infected TOCS until 72 hpi.

### RNA-seq analysis of TOCS samples

The number of raw reads generated after RNA sequencing of the TOCS samples varied from 53.6 million to 70.1 million (average ~60 million). Quality assessment of raw reads conducted using FastQC indicated PHRED scores > 30 for all samples and an average read length of 79 bp. Less than 1% of the reads were eliminated during trimming with Cutadapt before they were mapped separately to the chicken reference genome or to the CSW-1 ILTV reference genome with RNASTAR. The percentage of total mapped reads varied from 69.3% to 73.7%, with an average of 72% (Table S1).

### Identification of differentially expressed genes

**FIG 2:**
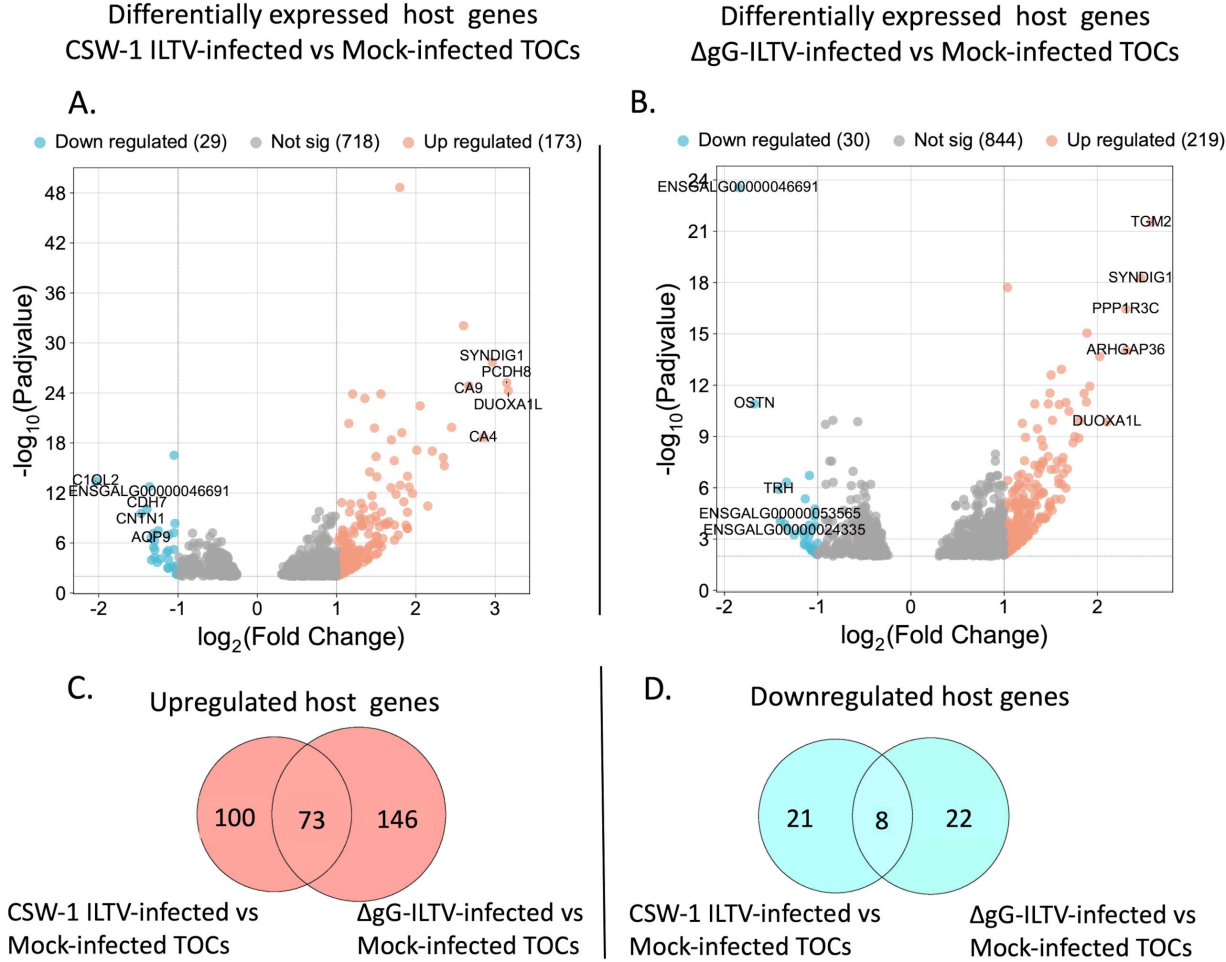
Volcano plots (**A and B**) representing the differentially expressed host genes in the (**A**) CSW-1 ILTV-infected TOCs compared with the mock-infected TOCs and (**B**) ∆gG-ILTV-infected TOCs compared with the mock-infected TOCs. Differences in the expression levels of host genes at *P*_adj_ value < 0.01 and fold change in expression greater than 2 (log_2_FC ≥ 1 = upregulated and log_2_FC ≤ −1 = downregulated) were considered statistically significant. The positions of the top 5 significantly up- or downregulated genes are labeled with gene symbols. Proportional Venn diagrams (**C**) and (**D**) represent the number of differentially expressed host genes in the CSW-1 ILTV and ∆gG-ILTV-infected TOCs compared with the mock-infected TOCs, (**C**) Upregulated host genes. (**D**) Downregulated host genes. The numbers in the intersection of the circles represent the commonly up- or downregulated genes, while the numbers outside of the intersections in the circles represent the uniquely up- or downregulated genes after infection with CSW-1 ILTV or ∆gG-ILTV compared with the mock-infected TOCs, respectively.

Differential gene expression analysis using DESeq2 identified the genes that were differentially regulated in the TOCs at 24 hpi with CSW-1 ILTV or ∆gG-ILTV compared with the mock-infected TOCs. Global gene expression patterns of the CSW-1 ILTV and ∆gG-ILTV-infected TOCs were different from each other as well as from the mock-infected TOCs and formed distinct clusters on the principal component analysis (PCA) plot (Fig. S1). Differences in expression levels of genes at *P*_adj_ value <0.01 and fold change greater than 2 (log_2_FC ≥ 1 = upregulated and log_2_FC ≤ −1 = downregulated) were considered significant when comparing either CSW-1 ILTV-infected or ∆gG-ILTV-infected TOCs to the mock-infected TOCs (Tables S3 to S6). More genes were upregulated than downregulated after infection with either strain of ILTV. The expression of 173 and 219 host genes was upregulated, while the expression of 29 and 30 genes was downregulated in the CSW-1 ILTV and the ∆gG-ILTV-infected TOCs, respectively, compared with the mock-infected TOCs (Fig. 2A and B). Log_2_FC values for the upregulated genes varied from 3.16 to 1.00 and 2.58 to 1.00, while that of the downregulated genes varied from −2.039 to −1.013 and −1.85 to −1.00 in the CSW-1 ILTV TOCs and the ∆gG-ILTV TOCs, respectively. Both ILTV-infected TOCs had 73 upregulated genes and 8 downregulated genes in common (Fig. 2C and D).

DESeq2 analysis was also performed for the identification of differentially regulated viral genes in the CSW-1 ILTV and the ∆gG-ILTV TOCs (Fig. S2A). However, except for the gene encoding gG (upregulated in the CSW-1 ILTV TOCs), the expression of no other ILTV genes was differentially regulated between the TOCs at the cut-off values (*P*_adj_ value < 0.01 and FC > 2) (Fig. S2B).

### Top 10 up- or downregulated host genes

The top 10 upregulated genes (Table 1) were involved in functions related to immune response, cell adhesion, transport, and lipid and glycogen metabolism. Among these, six were common to infection with CSW-1 ILTV or ∆gG-ILTV. Interestingly, a few hypoxia-induced genes (DUOXA1L, CA9, CA4, PPP1R3C) were present in the top 10 category of upregulated genes. The majority of the top 10 downregulated genes (Table 2) in the CSW-1 ILTV TOCs were associated with functions related to neuronal development. The others included genes encoding an adhesion molecule and a solute transporter. Several of the downregulated genes in the ∆gG-ILTV-infected TOCs were unannotated, including two genes that were also downregulated in the CSW-1 ILTV TOCs.

**TABLE 1:** Top 10 genes upregulated at 24 h post inoculation of TOCs with CSW-1 ILTV or ∆gG-ILTV compared with mock-infected TOCs^*a*^

Gene name (gene symbol) Function	Log_2_FC	
	CSW-1 ILTV	∆gG- ILTV
Dual-oxidase maturation factor 1-like (*DUOXL1*)^*b*^ Mostly expressed in airway epithelial cells. Involved in mucosal innate immune response via reactive oxygen species-mediated signaling (35). Plays a major role in calcium signaling during T cell activation following stimulation of avian erythrocytes (36).	3.17	2.11
ProTOCs adherin 8 (*PCDH8*) Calcium-dependent cell adhesion molecule. Involved in cell proliferation, migration, differentiation, and neuronal growth (37).	3.14	N
Synapse differentiation-inducing 1 (*SYNDIG1*)^*b*^ Regulates excitatory synapse development (38).	2.96	2.46
Carbonic anhydrase 4 (*CA4*) Expressed in cells subjected to hypoxic stress. O_2_/CO_2_ exchange in erythrocytes (39).	2.86	N
Carbonic anhydrase 9 (*CA9*)^*b*^ Expressed in cells subjected to hypoxic stress. Catalyze CO_2_ hydration reaction (40). Upregulation of CA9 in hypoxic mouse tumor cells was shown to induce adaptive immune response, dependent on IL-2 expression (41).	2.67	1.92
Lipase member M-like 5 (*LIPML5*) Role in avian lipid metabolism (42).	2.60	N
Transglutaminase 2 (*TGM2*)^*b*^ Regulation of cell death, cell survival, and activation of immune cells (43). Plays a vital role in dendritic cell activation, B cell differentiation, and CD8^+^ T cell generation during inflammation in mice (44). Overexpression of TGM2 in NDV-infected chicken embryos inhibited NDV replication via the activation of NF-κB signaling pathway and possibly through miRNA exocytosis- or membrane fusion-mediated IFN production, interfering with viral replication (45).	2.44	2.58
Rho GTPase-activating protein 36 (*ARHGAP36*)^*b*^ Cytoskeletal organization, growth, differentiation, neuronal development, and synaptic functions (46)	2.35	2.31
ST6 N-acetylgalactosaminide alpha-2,6-sialyltransferase 2 (*ST6GALNAC2*) Role in cell–cell communication, cell–substrate interaction, adhesion, etc. (47).	2.34	N
Suppressor of cytokine signaling 1(*SOCS1*)^*b*^ Inhibit interferon and NF-κB pathway induction (48, 49). Upregulated levels of SOCS1 were observed in both wild-type and vaccine ILTV infection *in vitro* in previous studies (13, 16) and at 2–4 days post Marek’s disease virus (MDV) infection in chickens (50–52).	2.21	2.03
Protein phosphatase 1 regulatory subunit 3C (*PPP1R3C*) Induced by hypoxia and regulates glycogen synthesis as a metabolic adaptation to hypoxic conditions (53). Upregulated in respiratory viral infections including SARS-COVID (54, 55). Role in glycogen metabolism in chickens during oxidative stress and hypoxia (56).	N	2.30
Ectonucleotide pyrophosphatase/phosphodiesterase 2 (*ENPP2*), Autotaxin Upregulates lysophosphatidic acid (LPA), which in turn promotes cell proliferation and immune cell chemotaxis, and hence implied in the pathogenicity of respiratory diseases in humans. Also involved in wound healing after tissue damage (57, 58).	N	1.88
Dipeptidase 2-like (*DPEP2*) Acts as an inflammatory regulator of macrophages (59). Metabolizes leukotriene D4 to leukotriene E4 (60).	N	1.88
Solute carrier family 16 member 6 (*SLC16A6*) Monocarboxylic acid transporter (61, 62).	N	1.86

^*a*^ *P*_adj_ value < 0.01 and log_2_FC ≥ 1 were considered significant.

^*b*^ Genes upregulated in both CSW-1 ILTV and ∆gG-ILTV TOCs; N, not in the top 10 category of upregulated genes.

**TABLE 2:** Top 10 genes downregulated at 24 h post inoculation of TOCs with CSW-1 ILTV or ∆gG-ILTV compared with mock-infected TOCs^*a*^

Gene name (gene symbol) Function	Log_2_FC	
	CSW-1 ILTV	∆gG- ILTV
Complement component 1, q subcomponent-like 2 (*C1QL2*) Complement protein involved in the coordination and regulation of motor functions (63).	−2.04	N/A
Contactin 1(*CNTN1*)^*b*^ Neuronal cell adhesion molecule (64).	−1.47	−1.133
Cadherin 7 (*CDH7*) Calcium-dependent cell adhesion molecule. Regulation of neuronal growth during cranial motor neuron development in chickens (65).	−1.39	N
ENSGALG00000046691^*b*^,^*c*^	−1.36	−1.85
Aquaporin 9 (*AQP9*)^*b*^ A membrane channel belonging to the aquaglyceroporin subfamily. Transports water, glycerol, urea, and other solutes and involved in gluconeogenesis (66).	−1.34	−1.33
Dipeptidyl peptidase-like 6 (*DPP6*) A potassium voltage-gated channel accessory protein with neuronal functions (67).	−1.33	N
Adhesion G protein-coupled receptor D1 (*ADGRD1*) Cell adhesion and migration (68).	−1.31	N
Leucine zipper protein 2 (*LUZP2*) A transcription factor predominantly expressed in the central nervous system and is associated with neuroendocrine functions (69).	−1.308	N
Neural EGFL-like 2 (*NELL2*) A secretory glycoprotein with several functions in neural development (70).	−1.30	N
ENSGALG00000009603^*b*^^,^^*c*^	−1.30	−1.19
Osteocrin (*OSTN*) A secretory protein produced by osteoblasts that regulate bone growth (71)	N	−1.68
Thyrotropin-releasing hormone (*TRH*) Regulator of the hypothalamic-pituitary-thyroid axis. Plays a role in the maintenance and restoration of immune homeostasis (72).	N	−1.42
ENSGALG00000053565^*c*^	N	−1.40
ENSGALG00000024335^*c*^	N	−1.35
ENSGALG00000051520^*c*^	N	−1.33
Nuclear factor-erythroid 2-related factor 2 (*Nrf2*) A transcription factor that contributes to anti-inflammatory response through the antioxidant response element (ARE) signaling pathway (73, 74).	N	−1.25

^*a*^ *P*_adj_ value < 0.01 and fold change > 2 (log_2_FC ≤ −1) were considered significant.

^*b*^ Genes downregulated in both CSW-1 ILTV and ∆gG-ILTV TOCs.

^*c*^ Unannotated genes, N; not in the top 10 category of downregulated genes.

### Genes with immune-related functions were differentially regulated by the two strains of ILTV in TOCs

Host genes with immune-related functions that were differentially expressed following infection with either strain of ILTV were further investigated. Genes encoding cytokines, chemokines, or their receptors were not downregulated following infection with either of the ILTV strains, while distinct sets of cytokines, chemokines, and several other immune-related genes were upregulated in each TOCs, indicating differential regulation of host immune response by the two strains of ILTV (Table 3).

**TABLE 3:** Immune-related host genes commonly and uniquely upregulated at 24 h post inoculation of TOCs with CSW-1 ILTV or ∆gG-ILTV^*a*^

Commonly upregulated after inoculation with CSW-1 or∆gG-ILTV	Uniquely upregulated after inoculation with	
	CSW-1 ILTV	∆gG-ILTV
*Cytokines and chemokines*		
C-C motif chemokine ligand 19 C-C motif chemokine ligand 17 Adiponectin	Interleukin 8-like 1 Interleukin 7	C-C motif chemokine ligand 26 C-C motif chemokine 26-like Cytokine like 1 Leukocyte cell-derived chemotaxin 1
*MHC-related proteins*		
	MHC B-G antigen	
*Chemokine and cytokine receptors*		
Interleukin 31 receptor A	Interleukin 13 receptor subunit alpha 2	Colony-stimulating factor 2 receptor beta common subunit
*Immune cell receptors*		
TNF receptor superfamily member 18 Histamine receptor H3	TNF receptor superfamily member 4 TNF receptor superfamily member 25	Macrophage mannose receptor 1-like 4 Cysteinyl leukotriene receptor 1 Cysteinyl leukotriene receptor 2 Histamine receptor H1
*Immunoglobulin (Ig)/Ig receptors*		
IgGFc-binding protein-like		IgGF-binding domain-containing protein
*Immunoregulatory proteins*		
Suppressor of cytokine signaling 1 V-set domain containing T cell activation inhibitor 1-like GTPase, very large interferon inducible pseudogene Lymphocyte-activating 3	Interferon induced with helicase C domain 1 Interferon-induced protein with tetratricopeptide repeats 5	Suppressor of cytokine signaling 3 Leukocyte ribonuclease A-2
*Complement components*		
		Complement C3b/C4b receptor 1 Complement C7

^*a*^ *P*_adj_ value < 0.01 and log_2_FC ≥ 1 were considered significant.

### Gene ontology (GO) analysis

**FIG 3:**
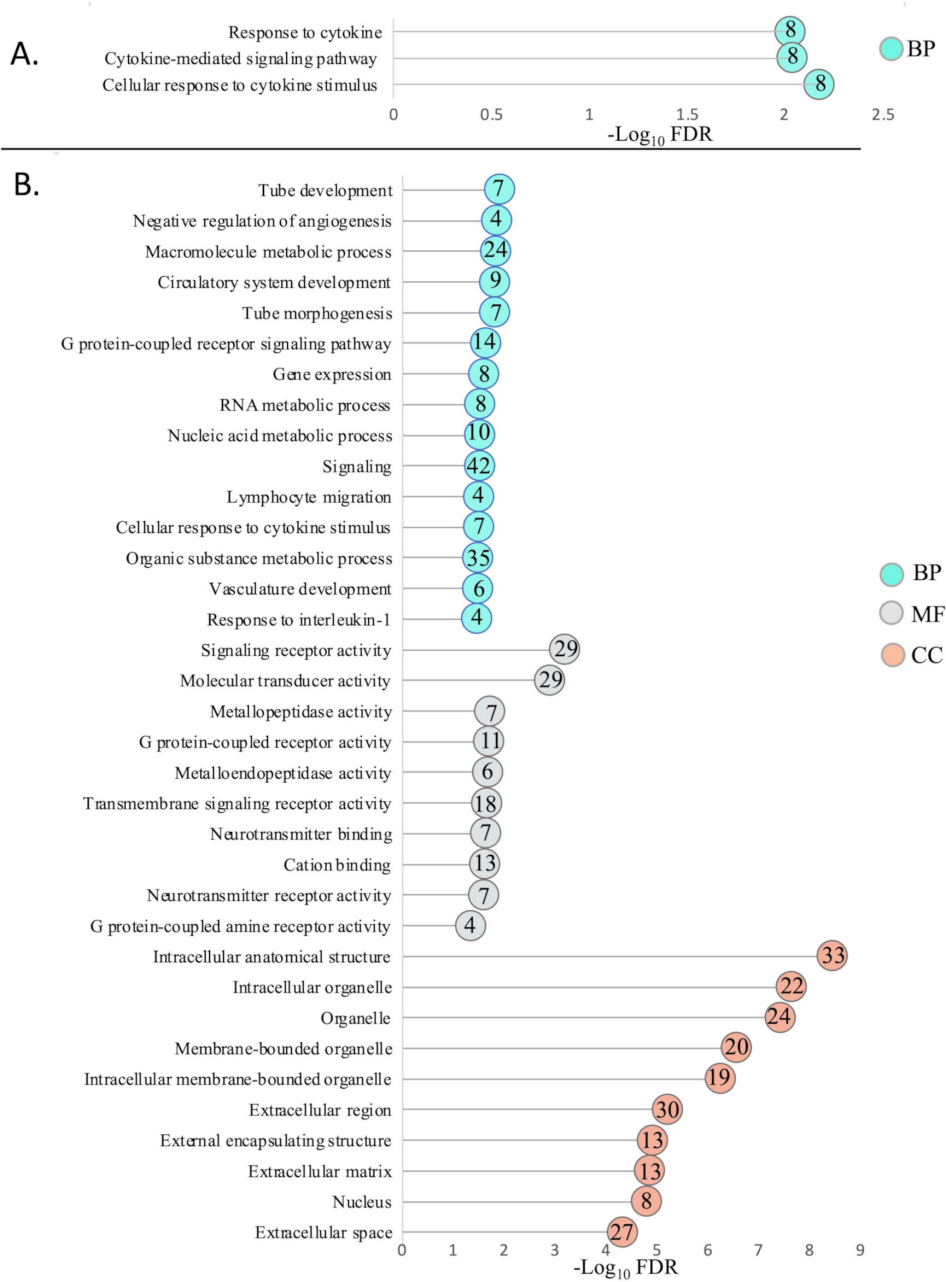
Gene ontologies (GO) most significantly enriched with the upregulated genes in the (**A**) CSW-1 ILTV or (**B**) ∆gG-ILTV-infected TOCs. Top 15 BPs, 10 MFs, and 10 CCs are listed. Only three GO terms were enriched for (**A**). The number of genes associated with each GO term is indicated in colored circles.

GO analysis using PANTHER identified the functional GO terms for biological processes (BPs), cellular components (CCs), and molecular functions (MFs) enriched with the genes up- or downregulated in the CSW-1 ILTV-infected or ∆gG-ILTV-infected TOCs. At a false discovery rate (FDR) < 0.05, 3 BP terms, but no CC or MF terms were enriched with the genes upregulated in the CSW- ILTV-infected TOCs while a total of 32, 10, and 17 GO terms for BPs, MFs, and CCs, respectively, were enriched with the upregulated genes of the ∆gG-ILTV-infected TOCs (Table S2). No GO terms were enriched with the genes downregulated in either of the ILTV-infected TOCs. The top 15 BP terms and top 10 each of the MF and CC terms upregulated in the CSW-1ILV-infected or ∆gG-ILTV-infected TOCs, arranged in the order of FDR, are shown in Fig. 3.

Upregulated BPs in the CSW-1 ILTV TOCs were involved in response to cytokine and cytokine signaling pathway while diverse BPs such as vasculature development, signaling, nucleic acid metabolism, cytokine-mediated signaling (including interleukin-1), as well as several terms related to chemotaxis, including lymphocyte, granulocyte, neutrophil (equivalent to chicken heterophils), and mononuclear cell migration, were enriched by the upregulated genes of the ∆gG-ILTV TOCs. The majority of the upregulated MFs were related signaling, neurotransmitter activity, and metallopeptidase activity while the CCs highlighted several terms related to both intracellular and extracellular components.

### Pathway analysis

At an FDR < 0.05, no Reactome or PANTHER pathways were enriched with the genes up- or downregulated after CSW-1 ILTV or ∆gG-ILTV infection of the TOCs.

### Protein classes

At FDR ≤ 0.023 and 0.033, one and six PANTHER protein classes were enriched with the genes upregulated in the CSW-1 ILTV and ∆gG-ILTV-infected TOCs, respectively, while no protein classes were enriched with the genes that were downregulated (Table 4). The protein class upregulated commonly between the CSW-1 ILTV-infected and ∆gG-ILTV-infected TOCs was that of “intracellular signal molecule” enriched with 12 (FDR = 0.022) and 13 (FDR = 0.013) genes each, respectively. Upregulated protein classes in the ∆gG-ILTV-infected TOCs included two classes related to extracellular matrix proteins, two classes involved in signaling, and one class each for cytokine and metalloprotease.

**TABLE 4:** PANTHER protein classes enriched with the genes upregulated in the TOCs infected with CSW-1 ILTV or ∆gG-ILTV compared with mock-infected TOCs

PANTHER protein	Number of upregulated genes	Fold enrichment	FDR^*a*^
*CSW-1 ILTV-infected TOCs*			
Intercellular signal molecule (PC00207)	12	3.77	2.23E-02
*∆gG-ILTV-infected TOCs*			
Extracellular matrix structural protein (PC00103)	12	12.4	9.67E-08
Extracellular matrix protein (PC00102)	16	7.78	1.66E-07
Transmembrane signal receptor (PC00197)	24	2.55	2.25E-03
Intercellular signal molecule (PC00207)	13	3.23	1.38E-02
Cytokine (PC00083)	5	7.66	2.72E-02
Metalloprotease (PC00153)	7	4.67	3.31E-02

^*a*^ A false discovery rate (FDR) < 0.05 was considered significant.

## DISCUSSION

This study assessed the differential response of host and viral genes 24 hpi with a wild-type (CSW-1 ILTV) parent strain of ILTV or a gG deletion mutant vaccine strain of ILTV (∆gG-ILTV) in TOC systems using RNA-seq analysis. In parallel, ILTV replication and localization in TOCs over time (until 72 hpi) were also evaluated. An increase in the amount of CSW-1 ILTV and ∆gG-ILTV detected in TOCs until 72 hpi was consistent with the observations of Coppo et al., while the presence of functional cilia in tracheal epithelial cells (until 72 hpi) was consistent with both Coppo et al. and Reddy et al., who observed normal ciliary beating until 7 dpi (17) and 96 hpi (21) in ILTV-infected TOCs. This underscores the finding that ILTV replication by itself is not sufficient to impair epithelial cell ciliary functions in the trachea (17). Loss of cilia reported in recent *in vivo* studies (75, 76) could therefore be an impact of immune cell infiltration or, as reported by Butler et al., a property of ILTV strain as not all strains of ILTV result in ciliostasis of tracheal epithelial cells (77).

Significant differences were observed in host gene transcription between the TOCs infected with CSW-1 ILTV and ∆gG-ILTV (each compared with mock-infected TOCs) (Tables S3 to S6). These findings indicate that gG may play a role in the modulation of the tracheal transcriptome during the early stages of ILTV infection, prior to infiltration by immune cells. The early effects of ILTV gG observed in this study are consistent with the kinetics of expression of this gene, which shares features of both immediate-early (IE) and late (L) genes (78). As a viral broad-spectrum chemokine-binding protein, it is hypothesized that gG may have a direct interaction with locally produced chemokines in the trachea. Additionally, it is possible that gG can also impact the expression and function of cytokines and chemokines downstream, thus leading to changes in the composition of the leukocyte milieu infiltrating the tracheal mucosa (17).

Of the genes differentially regulated after ILTV infection of TOCs, a total of 42.19% (73/173) and 33.33% (73/219) of the upregulated genes and 27.58% (8/29) and 26.66% (8/30) of the downregulated genes were in common between the CSW-1 ILTV-infected and the ∆gG-ILTV-infected TOCs, respectively. Host genes differentially regulated in the CSW-1 ILTV and ∆gG-ILTV- infected TOCs (compared with mock-infected TOCs) included several immune-related genes (Table 3). Upregulation of the genes for CR1 and C7 indicated the activation of complement systems in the ∆gG-ILTV-infected TOCs while the only complement-related gene that was differentially regulated in the CSW-1 ILTV TOCs was that of C1QL1, which was downregulated. While the transcription of cytokine or chemokine encoding genes was not downregulated in TOCs infected with either of the strains, upregulation of the genes for CCL17, CCL19, adiponectin, IL-7, IL8L1(chCXCLi1), CCL26, CCL26L, CYTL1, and LECT1 in the *ex vivo* systems indicated that these chemokines and cytokines were produced locally by resident cells in the tracheal microenvironment during ILTV infection.

Upregulation of the expression of adiponectin, CCL19, and CCL17 genes following infection with either of the ILTV strains in the TOCs in this study indicates that gG may not influence the transcription of these genes locally in the trachea. Of these chemokines, CCL19 and CCL17 were not found to interact with ILTV gG in a past study that assessed the affinity interactions of this vCKBP (gG) to a panel of human and mouse chemokines (27). While the involvement of these chemokines (CCL19 and CCL17) in ILTV immunity has not been demonstrated previously, upregulation of these genes *in vivo* has been observed during infection with other viruses (e.g., avian influenza virus and infectious bronchitis virus [IBV]) in chickens or other avian species (79–82). Upregulation of CCL19 in the trachea during ILTV infection may enhance T cell chemotaxis as this cytokine has been demonstrated to be a strong chemoattractant for chicken T cells during IBV infection (80).

Upregulation of genes encoding IL-7 and IL8L1 uniquely in the CSW-1 ILTV-infected TOCs and those for CCL26, CCL26L, CYTL1, and LECT1 uniquely in the ∆gG-ILTV-infected TOCs indicates that gG may impact their transcription in the *ex vivo* system. Increased transcription of IL-7 may contribute to interferon (IFN)-γ production and lymphocyte proliferation as seen during infectious bursal disease virus infection of chicken splenic tissues (83), while the upregulation of IL8L1 (84) may have a significant influence in the recruitment of immune cells during the acute phase of ILTV infection (17, 18). While no type I or type II IFN genes were differentially regulated in the CSW-1 ILTV or ∆gG-ILTV-infected TOCs, a few interferon-induced genes (IFIH1 and IFIT5) were upregulated in the former (Table 3). Although at a low FC, the upregulation of IL8L1 (log_2_FC = 1.02) in the CSW-1 ILTV-infected TOCs (but not in the ∆gG-ILTV- infected TOCs) in this study was different from the observations of Coppo et al., who noticed upregulation of the chCXCLi1 and chCXCLi2 genes in ∆gG-ILTV-infected TOCs (in the absence of gG) at 6, 24, and 48 hpi. Two alleles of CCL26 upregulated in the ∆gG-ILTV-infected TOCs may also have a role in immune cell recruitment *in vivo* as elevated levels of this chemokine (along with others) during the early phase of MDV infection in a recent study (79) were speculated to have a role in innate immune response and lymphoid cell migration to the site of infection in chickens. Taken in combination with the results of Devlin et al. that demonstrated high-affinity-binding interactions of ILTV gG to CCL26 (human) (equilibrium dissociation constant K = 2.6 × 10^−8^) (27), it can be inferred that gG of ILTV not only binds to CCL26, but its presence also appears to interfere with the transcription of this chemokine in tracheal mucosa. The other upregulated cytokine or chemokine genes, CYTL1 and LECT1, play a prominent role in the regulation of chondrogenesis (85, 86), and as such, their upregulation likely suggests modifications to the cartilaginous structure surrounding the trachea.

Modifications to structural components in the ∆gG-ILTV-infected TOCs were also highlighted by GO terms (Fig. 3) and protein classes (Table 4) enriched with the upregulated genes. This included several genes (Tenascin, Stabilin 1, Thrompspondin 1, Podoplanin, Connective tissue growth factor-like, Cas scaffold protein family member 4), upregulated uniquely in the ∆gG-ILTV-infected TOCs (not in CSW-1 ILTV-infected TOCs), with functions involved in immune cell proliferation and activation, and chemotactic migration of leukocytes, lymphocytes, and endothelial cells in humans or animal models (87–94). Furthermore, upregulation of macrophage mannose receptor 1-like 4 involved in lymphocyte trafficking (95), and cysteinyl leukotriene receptors (CYSLTR1 and CYSLTR2) (96–98) that enhance vascular permeability to leukocyte and eosinophil migration during airway inflammation in humans and mouse models, also indicated a distinct environment for immune cell migration in the trachea of the ∆gG-ILTV-infected TOCs compared with the CSW-1 ILTV-infected TOCs. These results were consistent with the suggested role of ∆gG-ILTV in modulating leukocyte migration to the site of infection (27) and warrant further investigations *in vivo.* Upregulation of several collagen genes (COL9A3, COL2A1, COL9A1, and COL28A1) in the ∆gG-ILTV-infected TOCs was different from the observations of Luo et al., who reported downregulation of collagen- and myosin-related genes in the trachea of chickens immunized with a different chicken embryo origin ILTV vaccine. While the implications of collagen gene upregulation in ∆gG-ILTV-infected TOCs in the current *ex vivo* study are not clear, the authors of the previous study (14) speculated that their downregulation may compromise the structure and function of the trachea. It is likely that the infiltration of leukocytes in the *in vivo* system compared with their absence in the *ex vivo* system may have driven tracheal tissue damage (17) in the chickens in the earlier study (14) as an increase in tracheal mucosal thickness resulting from leukocyte infiltration has also been observed after ∆gG-ILTV administration *in vivo* (29).

Interestingly, the expression of several immune checkpoint inhibitory genes such as SOCS1, SOCS3, LAG3, and VTCN1L was also differentially regulated in the ILTV-infected TOCs. Upregulation of genes encoding SOCS family of proteins has been reported during ILTV infection of chicken embryonic lung cells (13) and in MDV-infected chickens in multiple studies (50, 51), where chickens susceptible to MDV infection showed a greater upregulation of SOCS1 and SOCS3 genes (51). As SOCS1 (upregulated in both TOCs) and SOCS3 (upregulated in the ∆gG-ILTV-infected TOCs) inhibit type I and II interferon functions (99, 100), these genes (or their products) are susceptible to hijacking by a broad range of viruses including herpes simplex virus 1 (HSV-1) (100–104) to evade host immune surveillance and promote their replication. The existence of an immune evasion mechanism that impairs type I IFN response during ILTV infection was speculated by Vagnozzi et al., who suggested a role for ILTV genes ICP0, VP16, US11, or US3 in interfering with or blocking type I IFN signaling during ILTV infection, as seen during infection with HSV-1 (18). The viral and host protein interactions that resulted in the reduction of type I IFN signaling during ILTV infection, however, are not clear. Although speculative, the SOCS family members (upregulated in the current study) could interact with ILTV proteins to interfere with the IFN response as such interactions (HSV-1 ICP0-induced activation of SOCS1) during HSV-1 infection have shown to impair the IFN-γ response in human keratinocytes (100). *In vitro* studies utilizing SOCS1 and/or SOCS3 antagonists would be able to confirm the role of SOCS genes in type I IFN gene expression in chicken cells. Inducing T cell exhaustion through the expression of LAG3 (upregulated in both TOCs) has also been identified as an immune-evasion strategy of HSV-1 (105–107), while the expression of VTCN1L (also upregulated in both TOCs) is associated with the inhibition of T cell activation (108). Taken together, upregulation of these immune checkpoint inhibitors in this study indicated ILTV-mediated immune-evasion mechanisms. While the increased expression of SOCS genes may have a direct correlation to the downregulation of type I interferon expression associated with ILTV infection (17, 18), the upregulation of LAG3 and VTCN1L is consistent with the consensus that ILTV-mediated immune augmentation favors B cell responses over those of T cells (12, 22, 24, 25, 27, 28).

Local immune responses in the respiratory tract play a critical role in defense against respiratory viruses such as ILTV. Being the predominant site of ILTV replication, early host–virus interactions in tracheal mucosae are crucial in determining the outcome of ILTV infection. To gain a deeper insight into the molecular mechanisms underlying vaccine efficacy, future studies should focus on evaluating changes in transcriptome and protein expression during vaccination-challenge studies *in vivo*. This would include identifying the specific genes and pathways associated with ILTV antiviral immunity in chickens, including those vaccinated with ∆gG-ILTV. Such studies would contribute to a more comprehensive understanding of how the ∆gG-ILTV vaccine modulates the host’s response at the molecular level.

## MATERIALS AND METHODS

### Viral strains

This study used the following strains of ILTV: (i) an Australian virulent strain of ILTV, the CSW-1 ILTV; (ii) a gG-deficient attenuated vaccine strain of ILTV, the ∆gG-ILTV; and (iii) a fluorescent protein-tagged version of the CSW-1 ILTV strain, the m-cherry ILTV.

The ∆gG-ILTV strain was generated from the parent wild-type CSW-1 strain by homologous recombination by Devlin et al. (29). The m-cherry-tagged version of CSW1-1 ILTV was constructed according to the method of Russell et al. (109) using homologous recombination and CRISPR/Cas9 nuclease. A cassette containing the m-cherry gene between the CMV IE promoter and bovine growth hormone poly-A termination signal was inserted between the UL44 and UL21 genes of ILTV.CSW1. Overlapping complementary oligonucleotides corresponding to a UL44-UL21 intergenic site were cloned in the BbsI site of pX330 to act as guide RNA to target the Cas9 nuclease to the wild-type intergenic site (110). All three strains of ILTV were sourced from our laboratory and titrated on Leghorn male hepatoma (LMH) cells (111), as previously described (29).

### Collection of chicken trachea

Chicken tracheae for this experiment were sourced from five 6-week-old SPF chickens euthanized via intravenous administration of sodium barbiturate (Lethabarb, Virbac Australia). The trachea was exposed by making a scissor cut at the corner of the mouth and proceeding longitudinally down the neck until reaching the thoracic inlet. Exterior muscles and connective tissue surrounding the trachea were removed using sterile forceps and scissors, and the region of the trachea between larynx and thoracic inlet was excised and collected in TOCS medium (Dulbecco’s modified Eagle’s medium [DMEM, Merck KGaA, Darmstadt, Germany], 100 mg/mL ampicillin, 100 mg/mL gentamicin, 5 mg/mL amphotericin B, 10 mM HEPES [pH 7.7]) in sterile sample collection tubes (17).

### Preparation of TOCS

TOCs were prepared as described in previous studies (17, 112). Briefly, sterile phosphate-buffered saline (PBS) containing antimicrobials (100 µg/mL ampicillin, 5 µg/mL amphotericin B) was flushed through the lumen of each trachea three times to remove the accumulated mucus. Tracheae were then placed in a Petri dish flooded with TOCS medium. Using sterile forceps and a scalpel blade, each trachea was carefully cut into 1–2-mm-thick cross sections without collapsing the lumen. Tracheal rings were transferred to 24-well plates (three rings per well) containing 1.5 mL TOCS medium per well and observed under a light microscope to confirm ciliary movement of tracheal epithelial cells. Plates were left for overnight incubation on a plate shaker in a humidified environment in a cell culture incubator with 5% v/v CO_2_ in air at 37°C.

### Inoculation of TOCS

The following day, medium was removed from the wells and the tracheal rings were washed three times with sterile PBS to dislodge the mucus from the explants. Tracheal rings were then transferred to fresh 24-well plates (three rings per well) containing 1.5 mL TOCS medium per well and observed for ciliary movements using a light microscope. Prior to ILTV infection, medium was removed from the wells and nine wells each were infected with CSW-1 ILTV or ΔgG ILTV at 10^3^ plaque-forming units (PFUs) suspended in 0.5 mL of TOCS medium or mock-infected with 0.5 mL of TOCS medium. Three separate wells were also infected with 10^3^ PFU of m-cherry ILTV suspended in 0.5 mL for the detection of ILTV infection using fluorescence microscopy. After inoculation, the TOCs were left for viral adsorption on a plate shaker in the cell culture incubator (37°C in 5% v/v CO_2_) for 2 h. The inocula were removed after incubation and TOCs washed three times with 2 mL PBS containing antimicrobials and then replaced with 1.5 mL of fresh TOCS medium per well. The plates were gently swirled and then 500 µL of medium was immediately collected from each well to serve as the initial time point reference for ILTV detection and quantification in the TOCS supernatant. The plates containing the TOCs were then left for incubation (as described above) until each sampling time point. At 24, 48, and 72 hpi, TOCs were subjected to light microscopy to check for ciliary movements. Those that were infected with m-cherry ILTV were subjected to fluorescence microscopy to detect the localization of red fluorescence in the rings. After this, TOCS supernatant was collected from triplicate wells of the ILTV-infected or mock-infected TOCs and stored at −80°C for the detection and quantification of ILTV. Tracheal rings were collected in 600 µL RLT buffer (RNeasy Mini Kit, QIAGEN, Hilden, Germany) containing 1% (v/v) ß-mercaptoethanol and stored at −80°C until RNA extractions.

### Median TCID_50_ assay

Supernatant samples collected from ILTV-infected or mock-infected TOCs at 24, 48, and 72 hpi were subjected to TCID_50_ assay, as described previously (113). Briefly, 10-fold serial dilutions (up to 10^−8^) of the samples prepared in TOC titration medium (DMEM, 2% v/v fetal bovine serum [Sigma-Aldrich, Cat# 12003C-500mL, Buchs, Switzerland], 10 mM HEPES [pH 7.7], 100 µg/mL ampicillin, 100 µg/mL gentamicin, 5 µg/mL amphotericin B) were inoculated on LMH cells (passage 25) and incubated at 37°C and 5% v/v CO_2_ in air in a cell culture incubator. Cytopathic effects consistent with ILTV infection on LMH cells were observed until 72 hpi. Virus titer was determined using the Spearman and Kärber method and expressed as median TCID_50_/mL (114, 115).

### Nucleic acid extraction from TOCS supernatant samples

Supernatant samples collected from the TOCs at 24, 48, and 72 hpi were also subjected to nucleic acid extraction using MagMAX CORE Nucleic Acid Purification Kit (Applied Biosystems, Life Technologies Corporation, Austin, TX, USA) according to the manufacturer’s instructions. Extractions were performed using the automated high-throughput nucleic acid extraction instrument, the KingFisher Flex Purification System (Thermo Fisher Scientific). Then, 200 µL of supernatant samples were used as the starting material for nucleic acid extraction. Extractions were performed in 96-well plates, and nucleic acid was eluted into 90 µL of elution buffer each and stored at −80°C until quantitative real-time PCR (qPCR) assay.

### ILTV UL-15 qPCR assay

Nucleic acid samples were subjected to qPCR analysis for the detection and quantification of ILTV DNA. The SYBR green-based qPCR assay targeting the ILTV UL15 gene, developed by Mahmoudian et al. (116) and modified by Thilakarathne et al. (117), was used to determine the GCN of ILTV DNA in the supernatant samples. Standard curves were generated using 10-fold serial dilutions of pGEM-T (Promega, Madison, CA, USA) plasmid containing the ILTV UL15 gene amplicon (113 bp) (116). The UL15 GCNs in the samples were calculated based on cycle threshold values (Ct) using the Rotorgene Q software. The lower limit of detection of the assay was 100 copies per reaction.

### RNA extraction

For RNA extraction, tracheal rings (three rings per sample) in RLT buffer (RNeasy Mini kit) were placed on a sterile Petri dish, cut open, and mucosae scrapped off into the buffer using sterile scalpel blades. The mucosae (approximately 30 mg/sample) in the RLT buffer (600 µL) were then homogenized using Discofix 3-way stopcocks (B. Braun, Melsungen, Germany) and subjected to RNA extraction using RNeasy Mini kit following the manufacturer’s instructions. Eluates containing RNA were subjected to DNase treatment using the Turbo DNA-free kit (Invitrogen, Carlsbad, CA, USA), cleaned, and concentrated using Zymo RNA clean and concentrator-25 (Zymo Research Corporation, Irvine, CA, USA) according to the manufacturer’s instructions and stored at −80°C. The quality of total RNA was assessed using the Agilent 4200 TapeStation system (Agilent Technologies, Santa Clara, CA, USA) and all RNA samples were verified to have an RNA integrity number > 9.

### cDNA library preparation and RNA sequencing

RNA extracted from triplicate TOCS wells, harvested at 24 hpi, were subjected to cDNA library preparation for Illumina sequencing using a TrueSeq Stranded mRNA library preparation kit (Illumina Inc., San Diego, CA, USA) following the manufacturer’s recommendations. Briefly, poly-A-containing mRNA was isolated from 400 ng of total RNA from each replicate using poly-T-oligo magnetic beads. Purified mRNA was then fragmented and reverse-transcribed to produce first-strand cDNA using random primers and PCR amplification. This was followed by second-strand cDNA synthesis using DNA polymerase I and RNase H. The 3' ends of cDNA were then adenylated and subjected to single-index adaptor ligation. The end products were purified and enriched by PCR for the generation of the final cDNA libraries. The quality of the cDNA libraries was assessed using the Agilent 4200 TapeStation system (Agilent Technologies, Santa Clara, CA, USA) and all samples conformed to the required fragment size (~260 bp) for sequencing. Libraries were then normalized to 1 nM, pooled, denatured, and sequenced on a NextSeq500 sequencing platform (Illumina Inc., San Diego, CA, USA) using 80 bp paired-end approach.

### RNA-seq data preprocessing, quality control, and read mapping

Bioinformatics analyses of RNA-seq data for this experiment were conducted on the web-based analysis and workflow platform “GALAXY,” following the published RNA-seq data analysis workflow at usegalaxy.org.au (118–120). Raw files generated by Illumina sequencing (Illumina Inc.) were uploaded to the public server at usegalaxy.au for data analysis. Quality assessment of the raw reads was performed using FastQC version 0.11.8 (Galaxy version 0.72 + galaxy1). Reads in pairs were subjected to trimming using Cutadapt version 3.4 (Galaxy version 3.4 + galaxy1). Bases with PHRED quality score >20 and reads >20 bp in length were selected for downstream analysis. Ensemble database release 104 of the annotated chicken (*Gallus gallus*) genome (gene transfer format [gtf] and FASTA format) and the CSW-1 ILTV genome (GenBank accession number JX646899) were used as references to perform splice-aware mapping of the host and ILTV reads respectively with RNASTAR version 2.7.8 (Galaxy version 2.7.8a + galaxy0). Read summarization of BAM files (RNASTAR output) was performed using featureCounts subread version 2.0.1 (Galaxy version 2.0.1 + galaxy1) to generate exon-level read counts. Fragments (pairs of reads) with a minimum mapping quality score less than 10 and/or reads that align to multiple or overlapping features were excluded from analysis.

### Differential gene expression analysis

Differential gene expression analysis was performed using DESeq2 (121) version 1.22.1, Galaxy version 2.11.40.6 + galaxy2. Differences in the expression levels of host genes in the ILTV-infected TOCs compared with the mock-infected TOCs and ILTV genes in the ILTV-infected TOCs compared with each other at *P*_adj_ value (*P* values of Wald test corrected for multiple testing using the Benjamini and Hochberg method) <0.01 and with greater than 2-fold change in expression levels (log_2_FC ≥ 1 = upregulated and log_2_FC ≤ −1 = downregulated) were considered significant.

### Gene ontology, pathway, and protein class analysis

Identification of GOs, pathways, and protein classes enriched with the up- or downregulated genes was performed using PANTEHR (version 17.01) classification tool (122, 123). GOs, pathways, and protein classes enriched in the Reactome database within PANTHER at an FDR < 0.05 were considered significant and reported in this study.

### Statistical analysis

One-way analysis of variance with Tukey’s adjustment for multiple comparisons was used to test for differences in viral titers (TCID_50_ assay) and ILTV GCNs (UL15 qPCR assay) using GraphPad Prism (version 10). Data visualization was performed using R version 4.0, SRPLOT, and GraphPad Prism (version 10) software.

## Data Availability

RNA-seq data from this study is available at the National Centre for Biotechnology Information (NCBI), under the sequence reads archive (SRA) accession number PRJNA1074854.

## SUPPLEMENTAL MATERIAL

Tables S1 and S2 spectrum.01183-24-s0001.docx Table S1: Summary of reads. Table S2: Gene ontologies enriched with the upregulated genes in the ∆gG ILTV inoculated TOCs.

Tables S3 to S6 spectrum.01183-24-s0002.xlsx Datasets of upregulated and downregulated genes.

Figure S1 spectrum.01183-24-s0003.tif Principal component analysis (PCA) plot.

Figure S2 spectrum.01183-24-s0004.tif Principal component analysis (PCA) plot and volcano plot: ILTV.

ASM does not own the copyrights to Supplemental Material that may be linked to, or accessed through, an article. The authors have granted ASM a non-exclusive, world-wide license to publish the Supplemental Material files. Please contact the corresponding author directly for reuse.
