# Supplementary material for: Host–virus interactions during infection with a wild-type ILTV strain or a glycoprotein G deletion mutant ILTV vaccine strain in an ex vivo system
Source: Microbiol Spectr. 2025 Jan 13;13(2):e01183-24. doi: 10.1128/spectrum.01183-24 (PMC11792554; doi:10.1128/spectrum.01183-24)
Supplement: Tables S1 and S2 — Table S1: Summary of reads. Table S2: Gene ontologies enriched with the upregulated genes in the ∆gG ILTV inoculated TOCs. [file spectrum.01183-24-s0001.docx]

**Table S1** Summary of the reads from the TOCs 24 hours post mock inoculation, or inoculation with CSW-1 ILTV or ∆gG ILTV mapped to the chicken or ILTV genome.

| Group | Total mapped reads in million  (%) | Reads mapped to chicken genome  (%) | Reads mapped to ILTV genome  (%) |
| --- | --- | --- | --- |
| Mock_1^¶^ | 50.8  (73.7) | 50.8  (73.7) | 0 |
| Mock_2^¶^ | 52.8  (73.7) | 52.8  (73.7) | 0 |
| Mock_3^¶^ | 40.2  (72.8) | 40.2  (72.8) | 0 |
| CSW-1_1 ^*^ | 42.9  (70.1) | 37.6  (61.5) | 5.3  (8.6) |
| CSW-1_2 ^*^ | 45.6  (69.3) | 41.7  (63.3) | 3.9  (6) |
| CSW-1_3 ^*^ | 44.8  (71.3) | 38  (60.7) | 6.8  (10.6) |
| ∆gG^_^1 ^†^ | 49.9  (73.4) | 49.5  (72.8) | 0.4  (0.6) |
| ∆gG_2 ^†^ | 47.4  (72.6) | 46.9  (71.8) | 0.5  (0.8) |
| ∆gG_3 ^†^ | 39.6  (72.1) | 38.6  (70.2) | 1  (1.9) |

^¶^Replicates of the Mock-infected TOC group, ^*^Replicates of the CSW-1-ILTV-infected TOC group, ^†^Replicates of the ∆gG-ILTV-infected TOC group

**Table S2**: Gene ontologies enriched with the upregulated genes in the ∆gG ILTV inoculated TOCs compared to mock inoculated TOCs at 24 hours post inoculation.

| **GO category** | **Number of upregulated genes** | **Fold**  **enrichment** | **P-value** | **FDR** |
| --- | --- | --- | --- | --- |
| **PANTHER GO-Slim Biological Process** | | | | |
| tube development (GO:0035295) | 7 | 8.39 | 3.43E-05 | 1.27E-02 |
| negative regulation of angiogenesis (GO:0016525) | 4 | 36.75 | 1.30E-05 | 1.44E-02 |
| macromolecule metabolic process (GO:0043170) | 24 | 0.5 | 3.42E-05 | 1.52E-02 |
| circulatory system development (GO:0072359) | 9 | 6.1 | 2.82E-05 | 1.56E-02 |
| tube morphogenesis (GO:0035239) | 7 | 9.04 | 2.20E-05 | 1.62E-02 |
| G protein-coupled receptor signaling pathway (GO:0007186) | 14 | 3.49 | 7.55E-05 | 2.39E-02 |
| gene expression (GO:0010467) | 8 | 0.29 | 1.15E-05 | 2.55E-02 |
| RNA metabolic process (GO:0016070) | 8 | 0.34 | 2.35E-04 | 3.07E-02 |
| nucleic acid metabolic process (GO:0090304) | 10 | 0.37 | 2.26E-04 | 3.13E-02 |
| signaling (GO:0023052) | 42 | 1.77 | 2.73E-04 | 3.18E-02 |
| lymphocyte migration (GO:0072676) | 4 | 15.75 | 2.06E-04 | 3.26E-02 |
| cellular response to cytokine stimulus (GO:0071345) | 7 | 6.09 | 2.23E-04 | 3.30E-02 |
| organic substance metabolic process (GO:0071704) | 35 | 0.6 | 2.72E-04 | 3.35E-02 |
| vasculature development (GO:0001944) | 6 | 7.75 | 1.88E-04 | 3.48E-02 |
| response to interleukin-1 (GO:0070555) | 4 | 15.75 | 2.06E-04 | 3.51E-02 |
| regulation of vasculature development (GO:1901342) | 4 | 16.54 | 1.75E-04 | 3.52E-02 |
| mononuclear cell migration (GO:0071674) | 4 | 13.78 | 3.24E-04 | 3.59E-02 |
| regulation of angiogenesis (GO:0045765) | 4 | 16.54 | 1.75E-04 | 3.87E-02 |
| signal transduction (GO:0007165) | 39 | 1.8 | 3.73E-04 | 3.94E-02 |
| cytokine-mediated signaling pathway (GO:0019221) | 7 | 6.43 | 1.63E-04 | 4.02E-02 |
| regulation of multicellular organismal process (GO:0051239) | 10 | 3.79 | 4.31E-04 | 4.16E-02 |
| response to cytokine (GO:0034097) | 7 | 5.46 | 4.18E-04 | 4.21E-02 |
| granulocyte chemotaxis (GO:0071621) | 4 | 12.25 | 4.84E-04 | 4.30E-02 |
| primary metabolic process (GO:0044238) | 33 | 0.6 | 5.09E-04 | 4.34E-02 |
| blood vessel development (GO:0001568) | 6 | 8 | 1.60E-04 | 4.45E-02 |
| neutrophil migration (GO:1990266) | 4 | 12.25 | 4.84E-04 | 4.48E-02 |
| nitrogen compound metabolic process (GO:0006807) | 31 | 0.6 | 5.56E-04 | 4.57E-02 |
| cell communication (GO:0007154) | 41 | 1.73 | 6.40E-04 | 4.90E-02 |
| cellular macromolecule metabolic process (GO:0044260) | 21 | 0.53 | 7.12E-04 | 4.94E-02 |
| granulocyte migration (GO:0097530) | 4 | 11.03 | 6.94E-04 | 4.97E-02 |
| organelle organization (GO:0006996) | 7 | 0.34 | 6.28E-04 | 4.97E-02 |
| angiogenesis (GO:0001525) | 5 | 7.66 | 6.90E-04 | 5.10E-02 |
| **PANTHER GO-Slim Molecular Function** | | | | |
| signaling receptor activity (GO:0038023) | 29 | 2.65 | 2.43E-06 | 6.60E-04 |
| molecular transducer activity (GO:0060089) | 29 | 2.65 | 2.43E-06 | 1.32E-03 |
| metallopeptidase activity (GO:0008237) | 7 | 6.29 | 1.85E-04 | 2.01E-02 |
| G protein-coupled receptor activity (GO:0004930) | 11 | 3.79 | 2.26E-04 | 2.04E-02 |
| metalloendopeptidase activity (GO:0004222) | 6 | 7.19 | 2.75E-04 | 2.14E-02 |
| transmembrane signaling receptor activity (GO:0004888) | 18 | 2.71 | 1.66E-04 | 2.25E-02 |
| neurotransmitter binding (GO:0042165) | 7 | 5.51 | 3.96E-04 | 2.39E-02 |
| cation binding (GO:0043169) | 13 | 3.49 | 1.35E-04 | 2.45E-02 |
| neurotransmitter receptor activity (GO:0030594) | 7 | 5.57 | 3.75E-04 | 2.55E-02 |
| G protein-coupled amine receptor activity (GO:0008227) | 4 | 10.34 | 8.66E-04 | 4.70E-02 |
| **PANTHER GO-Slim Cellular Component** | | | | |
| intracellular anatomical structure (GO:0005622) | 33 | 0.42 | 7.51E-12 | 3.83E-09 |
| intracellular organelle (GO:0043229) | 22 | 0.36 | 9.50E-11 | 2.42E-08 |
| organelle (GO:0043226) | 24 | 0.38 | 2.34E-10 | 3.98E-08 |
| membrane-bounded organelle (GO:0043227) | 20 | 0.36 | 2.31E-09 | 2.94E-07 |
| intracellular membrane-bounded organelle (GO:0043231) | 19 | 0.36 | 5.83E-09 | 5.95E-07 |
| extracellular region (GO:0005576) | 30 | 3.07 | 7.53E-08 | 6.40E-06 |
| external encapsulating structure (GO:0030312) | 13 | 6.59 | 2.03E-07 | 1.29E-05 |
| extracellular matrix (GO:0031012) | 13 | 6.59 | 2.03E-07 | 1.48E-05 |
| nucleus (GO:0005634) | 8 | 0.25 | 2.90E-07 | 1.65E-05 |
| extracellular space (GO:0005615) | 27 | 2.91 | 9.74E-07 | 4.97E-05 |
| cytoplasm (GO:0005737) | 23 | 0.45 | 2.29E-06 | 1.06E-04 |
| intrinsic component of plasma membrane (GO:0031226) | 22 | 2.55 | 7.37E-05 | 3.13E-03 |
| integral component of plasma membrane (GO:0005887) | 21 | 2.47 | 1.64E-04 | 6.42E-03 |
| protein-containing complex (GO:0032991) | 13 | 0.42 | 1.84E-04 | 6.69E-03 |
| non-membrane-bounded organelle (GO:0043228) | 3 | 0.2 | 2.33E-04 | 7.44E-03 |
| intracellular non-membrane-bounded organelle (GO:0043232) | 3 | 0.2 | 2.33E-04 | 7.94E-03 |
| cell periphery (GO:0071944) | 61 | 1.45 | 1.83E-03 | 4.67E-02 |
