# Supplementary figures and images for: Host–virus interactions during infection with a wild-type ILTV strain or a glycoprotein G deletion mutant ILTV vaccine strain in an ex vivo system
Source: Microbiol Spectr. 2025 Jan 13;13(2):e01183-24. doi: 10.1128/spectrum.01183-24 (PMC11792554; doi:10.1128/spectrum.01183-24)

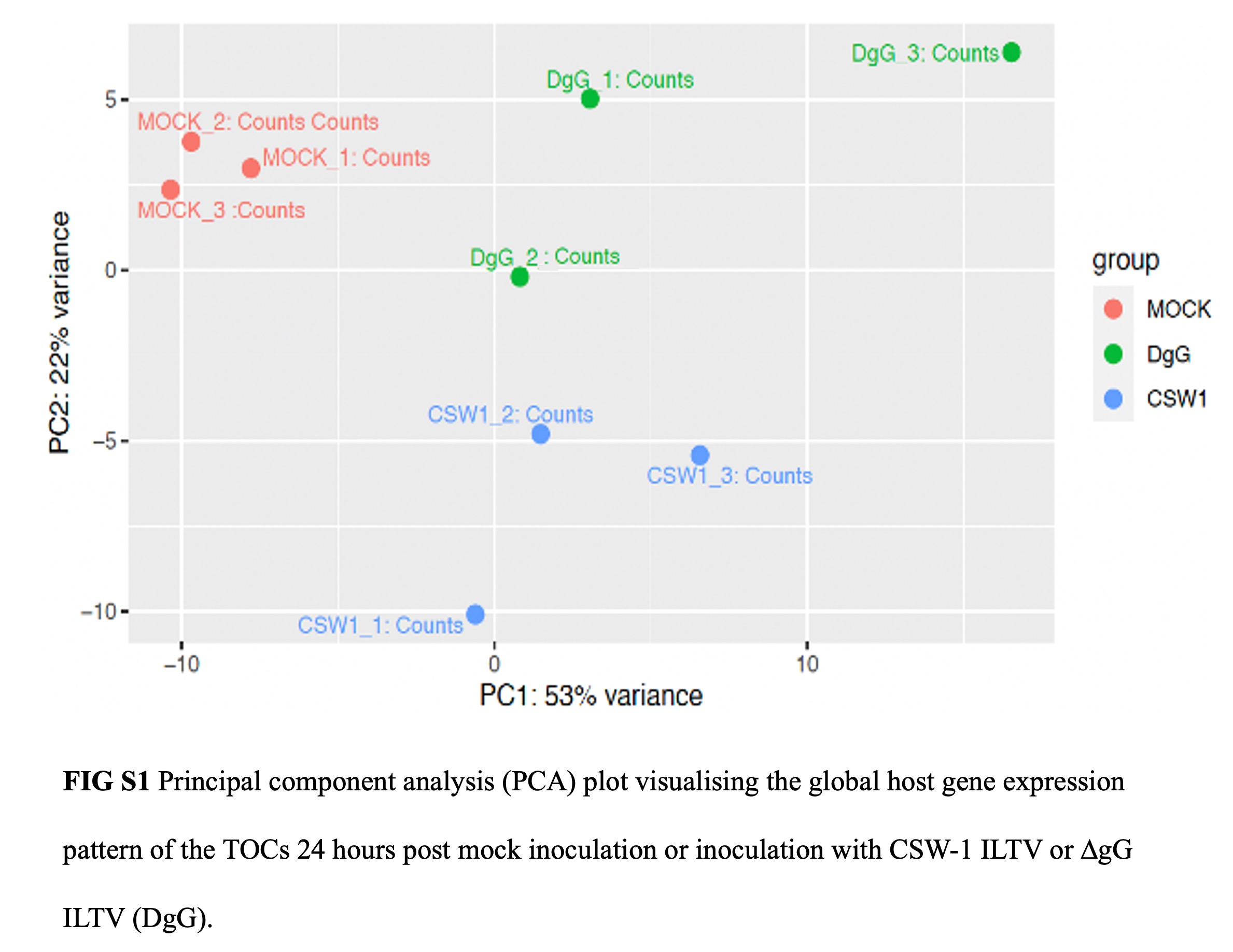

Supplement: Figure S1 — Principal component analysis (PCA) plot. [file spectrum.01183-24-s0003.tif]

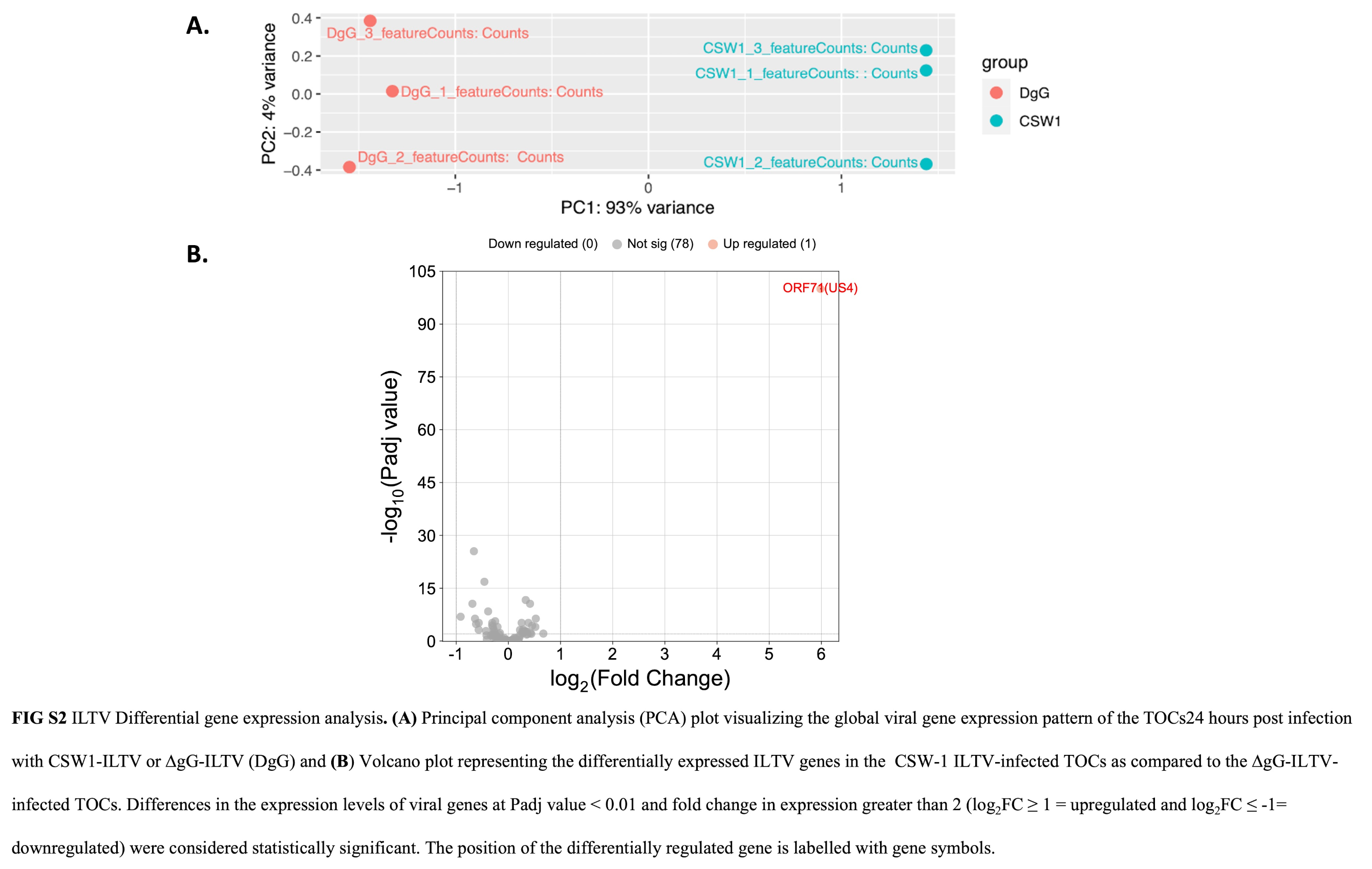

Supplement: Figure S2 — Principal component analysis (PCA) plot and volcano plot: ILTV. [file spectrum.01183-24-s0004.tif]
